# Complete genome sequence of *E. coli* lytic bacteriophage BAU.Micro_ELP-22, isolated from sewage water, Bangladesh

**DOI:** 10.1128/mra.00572-24

**Published:** 2024-09-09

**Authors:** Md. Arefin Kallol, K.H.M. Nazmul Hussain Nazir, Jahangir Alam, Md. Bahanur Rahman, Marzia Rahman

**Affiliations:** 1 Department of Microbiology and Hygiene, Faculty of Veterinary Science, Bangladesh Agricultural University, Mymensingh, Bangladesh; 2 National Institute of Biotechnology, Savar, Dhaka, Bangladesh; Department of Biology, Queens College, Queens, New York, USA

**Keywords:** *E. coli* phage, lytic bacteriophage, phage therapy, sewage wastewater

## Abstract

*Escherichia coli* lytic bacteriophage BAU.Micro_ELP-22 was isolated from sewage wastewater as a therapeutic agent alternative to antibiotics. The phage genome is 373,488 bp in length, encoding 744 protein-coding sequences and 7 tRNAs, and contains no antibiotic resistance, virulence, or temperate marker genes, which specifies its potentiality as a compatible phage therapy candidate.

## ANNOUNCEMENT

Antimicrobial resistance (AMR) is a critical menace worldwide. Phage therapy would be a striking hope to combat against AMR (1).

Here, we report the isolation, genome sequencing, and annotation of *Escherichia coli* lytic bacteriophage BAU.Micro_ELP-22 against multidrug-resistant avian pathogenic *E. coli* (APEC). The phage BAU.Micro_ELP-22 was isolated from untreated sewage wastewater in Mymensingh, Bangladesh. The collected water was centrifuged (12,000 *g*, 10 min), filtered using 0.22 µm syringe filter, and co-cultured with host bacteria (APEC environmental isolate code MR _VF-5, virulence factors- *rfbO157:H7*, *stx1*, *stx2*, *papC*, *fimC*, and *iucD*; provided by Professor Dr. Marzia Rahman, Department of Microbiology and Hygiene, Bangladesh Agricultural University) in Luria-Bertani broth for overnight at 37°C (150 rpm). The enriched sample was centrifuged (10,000 rpm, 10 min), and the supernatant was re-filtered (0.22 µm). Then, the phage BAU.Micro_ELP-22 was isolated from the filtrate, propagated, and purified (three rounds) through double-layer plaque assay (1) after spot test screening (2). Genomic DNA was extracted using the phenol-chloroform DNA extraction method (3). Illumina TruSeq Nano DNA Library Prep kit was used for preparing DNA library, and the sequencing was performed on Illumina NovaSeq 6000 sequencing system with paired-end reads of an average length of 150 nucleotides. Raw read quality was assessed using FastQC (Galaxy Version 0.74) (4). Next-generation sequencing reads were trimmed with trimmomatic (Galaxy Version 0.38.1) (5), and the genome assembly was performed with SPAdes (Galaxy Version 3.15.4) (6) with the parameters: operation mode—only-assembler and pipeline options—careful. Assembly validation was performed with the manual checking of contigs and the contigs read coverage (7). DNA termini and phage packaging mechanism were determined by PhageTerm (Galaxy Version 1.0.12) (8), and the genome assembly quality was evaluated with Quast (Galaxy Version 5.2.0) (9). Genome annotation was conducted using Pharokka (Galaxy Version 1.3.2) (10). Genome assembly and annotation completeness were assessed using BUSCO (Galaxy Version 5.5.0) (11). For all software, default parameters were used except where otherwise noted.

Genomic features have been represented in Table 1. No antimicrobial resistance, virulence factor, or temperate lifecycle encoding genes were found in the genome of phage BAU.Micro_ELP-22 analyzed with PhageLeads (12). According to PhageTerm analysis, the phage genome poses redundant ends termini and DRT class (direct terminal repeats) mode of packaging mechanism.

**TABLE 1:** Genomic features of phage BAU.Micro_ELP-22

Characteristic parameters	Description
Phage name	Escherichia phage BAU.Micro_ELP-22
Isolation sample	Sewage wastewater
Sampling location	24°44'51.5"N 90°25'22.2"E
Genome size (bp)	373,488 bp
GC content (%)	34.34
Coverage	181.389×
No. of genes	744
No. of genes with predicted function	156
No. of genes with hypothetical function	588
No. of tRNAs	7
No. of rRNAs	0
Number of CRISPRs	0
Lifestyle prediction	Lytic
Temperate marker genes	0
Presence of antibiotic resistance genes	0
Presence of virulence genes	0

*Escherichia* phage BAU.Micro_ELP-22 belongs to *Asteriusvirus* genus of *Caudoviricetes* bacterial virus class with head-tail morphology (NCBI: txid 3031943) (13). Blastp (https://www.ncbi.nlm.nih.gov) analysis of phage BAU.Micro_ELP-22 DNA polymerase protein (WPK29582.1) revealed 97.1%–100% similarities with the DNA polymerase protein of *Escherichia* phages ph0011, UE-S1, UB, and PBECO4 (accession no. WAE77201.1, UTS53925.1, AXC36763.1, and YP_009150826.1 orderly).

## Data Availability

The complete genome sequencing data for the BAU.Micro_ELP-22 phage are available in GenBank under the GenBank accession no. OR699282.2, Sequence Read Archive (SRA) accession no. SRR24112079, and Bioproject accession no. PRJNA941040. Purified BAU.Micro_ELP-22 phage high-titer lysates have been deposited at the Department of Microbiology and Hygiene Laboratory, Bangladesh Agricultural University, Bangladesh.
